# Effects of Natural Monoamine Oxidase Inhibitors on Anxiety-Like Behavior in Zebrafish

**DOI:** 10.3389/fphar.2021.669370

**Published:** 2021-05-13

**Authors:** Oihane Jaka, Iñaki Iturria, Marco van der Toorn, Jorge Hurtado de Mendoza, Diogo A. R. S. Latino, Ainhoa Alzualde, Manuel C. Peitsch, Julia Hoeng, Kyoko Koshibu

**Affiliations:** ^1^ Biobide, Gipuzkoa Scientific and Technological Park, San Sebastian, Spain; ^2^ PMI R&D, Philip Morris Products S.A., Quai Jeanrenaud 5, Neuchâtel, Switzerland; ^3^ Gestión de Recursos e Innovación S.L. (Grilab) C/ Américo Castro 94, Madrid, Spain

**Keywords:** alkaloids, harmane, norharmane, anxiety, zebrafish, monoamine oxidase, 1,2,3,4- tetrahydroisoquinoline, 2,3,6-trimethyl-1,4- naphtoquinone

## Abstract

Monoamine oxidases (MAO) are a valuable class of mitochondrial enzymes with a critical role in neuromodulation. In this study, we investigated the effect of natural MAO inhibitors on novel environment-induced anxiety by using the zebrafish novel tank test (NTT). Because zebrafish spend more time at the bottom of the tank when they are anxious, anxiolytic compounds increase the time zebrafish spend at the top of the tank and vice versa. Using this paradigm, we found that harmane, norharmane, and 1,2,3,4-tetrahydroisoquinoline (TIQ) induce anxiolytic-like effects in zebrafish, causing them to spend more time at the top of the test tank and less time at the bottom. 2,3,6-trimethyl-1,4-naphtoquinone (TMN) induced an interesting mix of both anxiolytic- and anxiogenic-like effects during the first and second halves of the test, respectively. TIQ was unique in having no observable effect on general movement. Similarly, a reference MAO inhibitor clorgyline—but not pargyline—increased the time spent at the top in a concentration-dependent manner. We also demonstrated that the brain bioavailability of these compounds are high based on the *ex vivo* bioavailability assay and in silico prediction models, which support the notion that the observed effects on anxiety-like behavior in zebrafish were most likely due to the direct effect of these compounds in the brain. This study is the first investigation to demonstrate the anxiolytic-like effects of MAO inhibitors on novel environment-induced anxiety in zebrafish.

## Introduction

Historically, plants have been a rich source of nutrients and chemical ingredients that help maintain physical and mental health for centuries (Perry and Perry, 2018). Scientific interests in these natural sources of health benefits have been steadily rising, where, for example, investigations on “herbal medicine” alone sored from approximately 2,000 publications in 2010 to almost 5,000 publications in 2020 reported on PubMed (accessed Feb 15, 2021). In some cases, active ingredients in plants have been identified and investigated for specific health benefits (Perry and Perry, 2018). Monoamine oxidase (MAO) inhibitors are a class of one such naturally occurring compounds that have been clinically developed as an antidepressant and as a treatment for social anxiety and Parkinson’s disease (Youdim et al., 2006; Finberg and Rabey, 2016; Menkes et al., 2016; Tipton, 2018; Sabri and Saber-Ayad, 2020). Monoamine oxidase is a widely distributed mitochondrial enzyme with high levels of expression in the brain as well as gastro-intestinal and hepatic tissues (Fowler et al., 2003; Finberg and Rabey, 2016). Two isoenzymes of MAO – MAO-A and MAO-B – are present in most mammalian tissues and can be differentiated by their substrate specificities, inhibitor sensitivities, and tissue localizations (Shih et al., 1999; Tipton, 2018). The enzyme catalyzes the oxidative deamination of a variety of monoamines, both endogenous and exogenous. It has major roles in metabolizing neurotransmitters, including serotonin, histamine, dopamine, noradrenaline, and adrenaline, and in detoxifying a large variety of endogenous and exogenous amines (Dos Santos Passos et al., 2014; Finberg and Rabey, 2016; Edmondson and Binda, 2018).

In nature, various plants or plant extracts, such as a chewing nut *Areca catechu* L. (Arecaceae) and a popular curry or tea component *Curcuma longa* L. (Zingiberaceae), have been identified to suppress MAO activity and induce anti-depressant-like effects (Vina et al., 2012). Similarly, the leaves of *Ginkgo biloba* L. (Ginkgoaceae) have been suggested to restore the striatal dopamine levels in rodent models of Parkinson’s disease through the regulation of MAO, although the antioxidant property of *G. Biloba* probably also contributed to the observed effects (Pardon et al., 2000; Ahmad et al., 2005). In addition, MAO inhibiting *β*-carbolines in *Coffea* (Rubiaceae) – more commonly known as coffee – have been suggested to induce neuroprotection against Parkinson’s disease (Herraiz and Chaparro, 2006). The effect of *β*-carbolines and other MAO inhibitors on anxiety, in contrast, seems to be rather complex, with early findings reporting anxiogenic effect in rhesus monkeys and humans (Schweri et al., 1982; Skolnick et al., 1984; Crawley et al., 1985), but withdrawing from MAO inhibitor treatments has been associated with anxiety (Dilsaver, 1994). In addition, MAO inhibitors seem to induce anxiolytic effect in specific types of anxiety, such as social phobia (Liebowitz et al., 1993; Schneier, 2001).

In this study, we aimed to understand the effect of natural MAO inhibitors on another behavioral phenomenon, novel environment-induced anxiety, in a relatively high throughput behavioral paradigm–the zebrafish novel tank test (NTT). During the past few decades, zebrafish have emerged as a model vertebrate organism for analyzing complex molecular and cellular interactions *in vivo* (Eliceiri et al., 2011; Stewart et al., 2012; Stewart and Kalueff, 2012; Pickart and Klee, 2014). Their usefulness as an animal model for neurobehavioral research has been recognized, and mounting evidence suggests the suitability of zebrafish for modeling various aspects of anxiety-related states (Stewart et al., 2012; Khan et al., 2017b; Kysil et al., 2017). The NTT takes advantage of the innate behavior of zebrafish to dive and dwell at the bottom of a body of water to avoid danger or stress. It is a relatively high-throughput behavioral test with some translational relevance to humans (Levin et al., 2007; Papke et al., 2012; Stewart et al., 2012). For example, anxiolytic drugs, such as diazepam and buspirone, have been shown to induce anxiolytic-like efficacy in this zebrafish behavioral paradigm (Levin et al., 2007; Bencan and Levin, 2008; Bencan et al., 2009; Stewart et al., 2012). Using this zebrafish model, three alkaloids (harmane, norharmane, 1,2,3,4-tetrahydroisoquinoline (TIQ)), and 2,3,6-trimethyl-1,4-naphtoquinone (TMN), previously identified to be present in, for example, solanaceous and rubiaceous plants (Boulton et al., 1988; Herraiz and Chaparro, 2006), were investigated in order to understand their potential efficacy in modulating anxiety-like behavior in animals. Buspirone, a clinically approved anxiolytic drug, was also tested to confirm the validity of the NTT. Harmane, norharmane, and TIQ were selected due to previous reports suggesting their role in regulating depression or anxiety in rodents and/or in humans (Pepplinkhuizen et al., 1996; Verheij et al., 1997; Aricioglu and Altunbas, 2003; Farzin and Mansouri, 2006; Smith et al., 2013; Antkiewicz-Michaluk et al., 2014; Mozdzen et al., 2014; Dos Santos et al., 2016; Khan et al., 2017a; Piechowska et al., 2019). Harmane and norharmane are the most abundant *β*-carbolines found in numerous plants and food stuffs (Herraiz, 2004, 2007; Piechowska et al., 2019). Passifloraceae flowers of plants belonging to this family (e.g., passion fruit) have been found to contain 126 ± 27 ng/g dry matter (d.m.) harmane and 68.3 ± 5.3 ng/g d.m. norharmane, as well as in herbal medicines, such as Evodiae Fructus (a.k.a., wu zhu yu; 0.63 ± 0.13 μg/g harmane and 8.24 ± 0.13 μg/g norharmane) and *Tribulus terrestris* (a.k.a., puncture vine; 44 μg/g d.m for both to 99.5 ± 13.2 ng/g and 131 ± 11 ng/g, respectively) (Tsuchiya et al., 1999; Pfau and Skog, 2004; Piechowska et al., 2019). In food, harmane and norharmane are found in, for example, brewed coffee (335 ± 105 ng/g and 1430 ± 540 ng/g ground coffee, respectively), soy sauce (187.6 ± 21.9 μg/L and 44.0 ± 10.6 μg/L, respectively), well-done cooked meat (26.4 ± 8.05 ng/g and 82.3 ± 64.8 ng/g, respectively) – but not in raw or medium cooked meat –, and raisins (6–644 ng/g and 2–120 ng/g, respectively) (Herraiz, 2004; Pfau and Skog, 2004; Herraiz, 2007). *β*-carbolines are reversible competitive inhibitors of MAO. In addition to its potent inhibitory activity particularly against MAO A, harmane has been reported to act on serotonin, opiate, dopamine, imidazole, benzodiazepine receptors as well as acetylcholinesterase and butyrylcholinesterase (Arib et al., 2010; Khan et al., 2017a). Norharmane inhibits both MAO A and B and also has been suggested to act on other targets similar to harman, such as serotonine, benzodiazepine, and opiate receptors (Pepplinkhuizen et al., 1996).

TIQ is an isoquinoline derivative found in foods with a high 2-phenylethylamine content, such as white wine (1.7 ± 0.8 ng/g), cheese (15.0 ± 2.2 ng/g), and cocoa (0.8 ± 0.3 to 1.1 ± 0.8 ng/g), and have been detected in rat and human brains (Makino et al., 1988). Isoquinoline alkaloids are a large family of phytochemicals found in plant families, such as Papaveraceae, Berberidaceae, and Ranunculaceae, and have been used in folk medicine as, for example, digestive stimulant, immune stimulant, muscle relaxant, analgesic, sedative, and anti-inflammatory (Khan and Suresh Kumar, 2015). Less information is available about TIQ, but it has been reported to reversibly inhibit MAO-A and B and acts as an anti-depressant in rodents (Thull et al., 1995; Antkiewicz-Michaluk et al., 2014; Mozdzen et al., 2014). It is an interesting compound, because it forms a basic chemical unit for numerous other natural isoquinolines and for drugs for relaxation, such as tubocurarine, and anti-depressants, such as diclofensine (Cherpillod and Omer, 1981; Exley et al., 2005).

Lastly, TMN was selected to understand a novel anxiolytic-like property of this compound that has been previously identified to be a MAO inhibitor isolated from a tobacco plant (Mostert et al., 2016). TMN is a derivative of naphthalene. Naphthoquinones are secondary metabolites derived from primary metabolites – carbohydrates, amino acids and lipids – that are present in various organisms, such as plants (e.g. Ebenaceae), fungi (e.g. *Fusarium* spp.), lichens (e.g. *Cetraria* spp.), algae (*Landsburgia quercifolia*), and in actinomycetes (*Streptomyces* spp.) (Babula et al., 2009; Pinho et al., 2012). One of more well-known members of this chemical class include vitamin K (Coelho Cerqueira et al., 2011; Pinho et al., 2012). The biological effects of naphthoquinones are wide and include neuroprotection, cardioprotection, and hepatoprotection as well as anti-inflammatory and anti-microbial activities (Aminin and Polonik, 2020). Thus, this chemical family is increasingly considered as a source of drug development (Babula et al., 2009; Pinho et al., 2012; Aminin and Polonik, 2020). It has been reported that TMN, a minor component of flue-cured tobacco leaves and smoke, is a reversible competitive inhibitor of MAO-A and MAO-B that exhibits protective properties against MPTP toxicity in mice (Castagnoli et al., 2001; Coelho Cerqueira et al., 2011). To our knowledge, the present study is the first to examine the effects of TIQ and TMN on anxiety-like behavior in a non-clinical animal model.

Furthermore, to support the validity of our behavioral findings, we demonstrated that these natural MAO inhibitors indeed reach the brain by using the *ex vivo* bioavailability assay and in silico blood brain barrier models.

## Materials and Methods

### Animals

Wild-type zebrafish (*Danio rerio*; strain AB) were bred and housed at Biobide (San Sebastián, Gipuzkoa, Spain) in accordance with standard procedures (Zebrafish Information Network) as described previously (Alzualde et al., 2018; Quevedo et al., 2019). In brief, the fish were maintained in a 300-L aquarium with a maximum of 1,000 fish per tank. The system water was maintained at 28.5°C, pH 7–7.8, 500–800 μS conductivity, and 80–100% oxygen and continuously filtered. The system water condition was monitored daily and regulated. The fish were kept under a 14-/10 h light/dark cycle (light on at 7:30 am). Adult fish were fed ground dry pellets (Gemma Micro 300; Sketting Zebrafish, Westbrook, ME, United States) and live food (artemia; Catvis B.V., ’s-Hertogenbosch, Netherlands) once a day. All behavioral experiments were performed on adult male and female zebrafish (approximately 36–52 weeks post fertilization) in accordance with European standards of animal welfare in animal use for scientific purposes (2010/63/EU), compiled with national regulations for the care of experimental animals, and were approved as described in national regulations (RD 53/2013) by local and regional committees: PRO-AE-SS-121 and PRO-AE-SS-134.

### Chemicals

**FIGURE 1:**
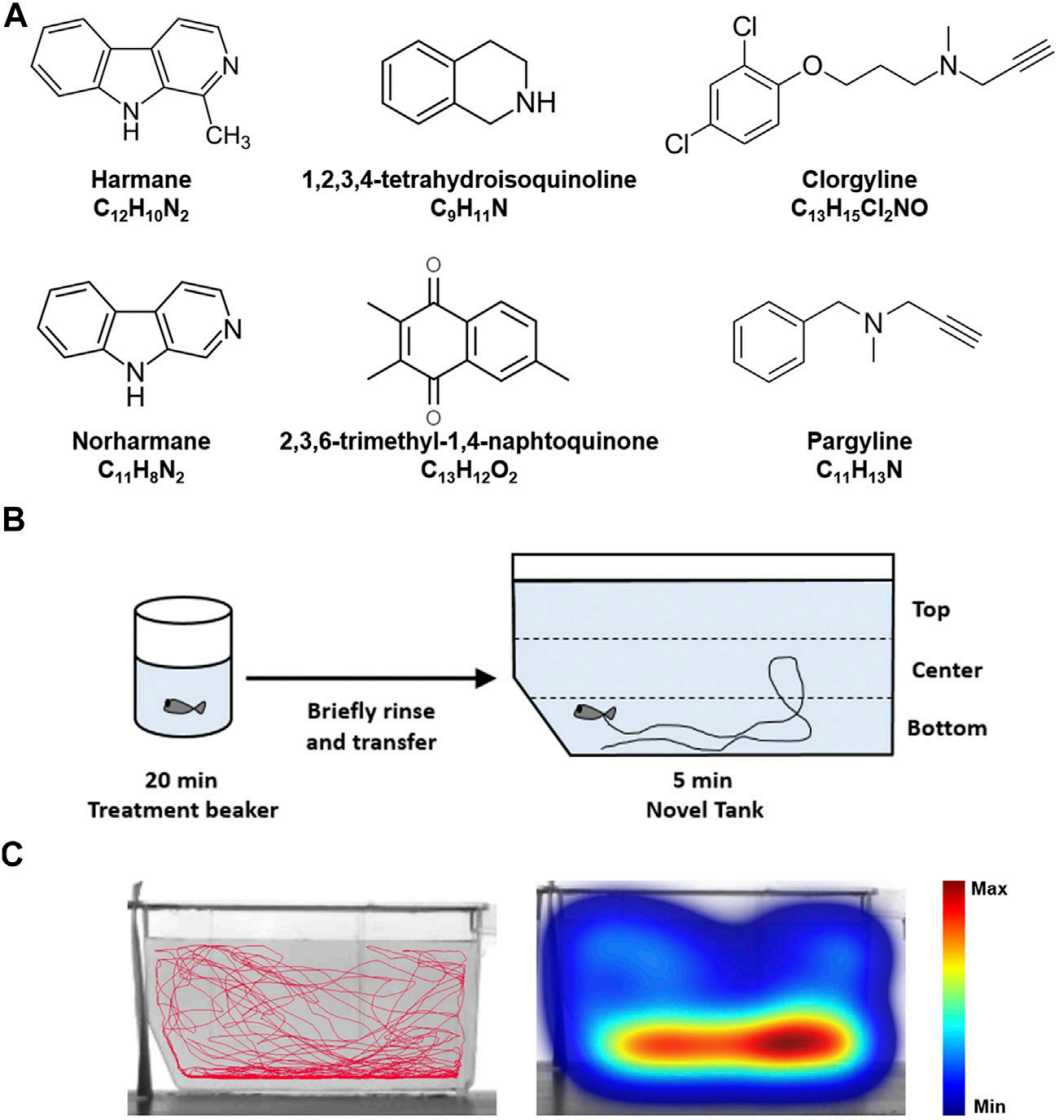
Zebrafish NTT experimental paradigm **(A)** The chemical structures of the test compounds–harmane, norharmane, TMN, TIQ, clorgyline, and pargyline–are shown **(B)** Zebrafish are placed in a treatment beaker with respective compounds for 20 min, briefly rinsed in system water, and then monitored in a novel tank for 5 min. The time spent at the top and bottom one third of the tank were then analyzed **(C)** A representative trace and heatmap of the swimming activity is presented for a vehicle control. The heatmap shows the minimum (dark blue) to maximum (dark red) amount of time a fish spent in each pixel. Abbreviations: NTT = novel tank test.

Harmane (CAS No. 486–84–0), norharmane (CAS No. 244–63–3), 1,2,3,4-tetrahydroisoquinoline (TIQ; CAS No. 91–21–4), clorgyline hydrochloride (CAS No. 17780–75–5), and pargyline hydrochloride (CAS No. 306–07–0) were purchased from Sigma-Aldrich® (St. Louis, MO, United States of America). 2,3,6-trimethyl-1,4-naphtoquinone (TMN; CAS No. 20490–42–0) was purchased from Enamine Ltd. (Kyiv, Ukraine). Buspirone hydrochloride (CAS No. 33386–08–2) was purchased from Tocris Bioscience (Bio-Techne®, Minneapolis, MN, United States). The chemical structures of compounds are shown in Figure 1A.

### Zebrafish NTT

Adult male and female wild-type zebrafish were treated with the compounds for 20 min in a final volume of 50 ml in a 250-ml treatment beaker, one fish at a time. The treatment concentrations were determined by first testing the compounds at 30 mg/L. If the fish tolerated the concentration in the treatment beaker for 20 min, then higher concentrations were tested. If not, the concentration was reduced until no obvious signs of tolerability problems (e.g., seizure-like tail or body movement) were observed. The test concentrations for the NTT were as follows: harmane (1, 3, and 10 mg/L; equivalent to 5, 17, and 55 µM), norharmane (0.3, 1, and 3 mg/L; equivalent to 2, 6, and 18 µM), TIQ (10, 30, and 100 mg/L; equivalent to 75, 226, and 752 µM), TMN (3, 10, and 30 mg/L; equivalent to 15, 50, and 150 µM), clorgyline (10, 30, and 100 mg/L; equivalent to 32, 97, and 324 µM), pargyline (10, 30, and 100 mg/L; equivalent to 51, 153, and 510 µM), and buspirone (10, 30, and 100 mg/L; equivalent to 26, 78, and 259 µM).

Following compound treatment in a 250 ml beaker, the fish were briefly rinsed in fresh system water, and then immediately transferred to a trapezoidal tank (14.6 cm height x 5.5 cm width x 27.9 cm top length and x 23.6 cm bottom length) filled with 1.5 L system water (Figure 1B). The behavior of the fish was monitored for the next 5 min by using the Noldus EthoVision XT system (Wageningen, Netherlands), with the camera placed approximately 1 m from the test tank (Figure 1C). The water-filled part of the tank (11.5 cm height) was virtually divided into top, center, and bottom (Figure 1B). The average time spent at the top and bottom portions of the tank was analyzed to determine the anxiety-like behavior of fish. The average total distance traveled and freezing time (as defined by a complete cessation of movement except for gills and eyes (Kalueff et al., 2013)) were calculated to determine the effects of the compounds on the general behavior of fish. The analyses were conducted in 1-min time bins to demonstrate the change in their behavior overtime. Any fish that stayed immobile for longer than 200 s out of a total of 5 min test period were considered as an outlier as they were generally >2 standard deviations away from the mean and excluded from all analysis. One fish from 10 mg/L harmane, one fish from 3 mg/L norharmane, one fish from 100 mg/L TIQ, two fish from 10 mg/L TMN, five fish from 30 mg/L TMN, four fish from 30 mg/L clorgyline, six fish from 100 mg/L clorgyline, and three fish from 100 mg/L buspirone were removed from the final analysis as their behavior was uninterpretable. There was no sex-dependent effect on these abnormally long bouts of freezing. Relatively high number of zebrafish that showed freezing for the highest concentrations of TMN, clorgyline, and buspirone, suggested that the respective concentrations were at the borderline of tolerability. The experimenters were blind to the test conditions. A minimum of 12 fish (6 females and 6 males) per condition were used for the study.

### Monoamine Oxidase Assay

The two-step bioluminescent assay (MAO-Glo™ Assay Systems; Promega, Madison, WI, United States) was performed in Nunc white, 96-well, flat-bottom assay plates (Life Technologies Europe B.V., Zug, Switzerland) as described previously (Van Der Toorn et al., 2019). Fluostar Omega 96 Microplate reader (BMG LABTECH GmbH, Ortenberg, Germany) was used to measure the luminescent signal and to determine the IC_50_ (half maximal inhibitory concentration). Z’ scores were between 0.75 and 0.86 for both MAO A and B assays. The constant (Km) of MAO A was 17.1 and 2.6 µM for MAO B. The substrate concentrations (S) were 20 µM for MAO A assay and 3 µM for MAO B. The Ki values referred in the discussion were calculated accordingly to the following formula: $Ki=IC_{50}/\left(1+\frac{Km}{S}\right)$.

### Brain Dissection

Four fish (2 male and 2 female) per test condition were exposed to 3 mg/L harmane, 3 mg/L norharmane, 30 mg/L TMN, 100 mg/L TIQ, 100 mg/L clorgyline, or 100 mg/L pargyline. The selected concentrations were either the highest effective concentration or the highest tested concentration to optimize the chance of compound detection in the brain. Immediately after the 20 min compound treatment, the zebrafish were rinsed briefly to remove excessive compound on their body and euthanized with 250 mg/L tricaine (CAS No. 886–86–2; Sigma). The fish were decapitated at the level of the gills by using a surgical knife. The head was turned dorsal side down, and, soft tissue was removed from the ventral side of the skull using two forceps until the base of the skull was exposed. The skull was broken open, and the bone from the ventral side of the brain was removed. The brain was then placed in a microcentrifuge tube, weighed, snap-frozen in liquid nitrogen, and stored at −80°C until the analysis.

### Bioavailability Assay

Briefly, on the day of analysis, the brain samples were defrosted, resuspended in a methanol solution (2:1 [v/v] methanol:MilliQ water), and homogenized with vigorous agitation and ultrasonication (5 min each). The homogenate was centrifuged at 15,000 x rpm for 10 min, and the supernatant was analyzed by using a UPLC-Q Exactive Orbitrap-HRMS system (Thermo Fisher Scientific™, Bremen, Germany) for all compounds except for TMN, which was analyzed by gas chromatography/mass spectrometry (GC/MS) system. Chromatographic separation was achieved on a Synergi™ 4-µm Hydro-RP 80 Å, L.C. Column (250 × 4.6 mm; Phenomenex Inc., Torrance, CA, United States) with 0.1% formic acid in water (mobile phase A) and 0.1% formic acid in acetonitrile (mobile phase B). A gradient method at a 500 µL/min flow rate was applied as follows: 1) 5% B for 1 min and 2) increase to 95% B over 7 min and hold for 2 min. The injection volume was 5 μL. The mass spectrometer was operated in electrospray positive mode (ESI; Thermo Fisher Scientific), while data acquisition was performed by using the parallel reaction monitoring (PRM) and full scan modes. The source settings were set as follows: sheath gas flow rate = 60 psi; aux gas flow rate = 20 arbitrary units; spray voltage = 3.5 kV; capillary temperature = 280°C; and sweep gas flow rate = 1. The full scan mode parameters were set as follows: resolution = 35,000 FWHM at 200 m/z; AGC target = 1E6; maximum injection time = 110 ms; and scan range = 100–350 m/z. The chromatographic and Orbitrap MS parameters for PRM analysis were the same as those in the full scan mode, except for: AGC target = 2E5; maximum IT = 60 ms; and resolution = 17,500 FWHM at 200 m/z. The XCalibur™ v4.0.27.19 software (Thermo Fisher Scientific) and TraceFinder™ v4.1 Forensic (Thermo Fisher Scientific, San José, CA, United States) were used for system control and data processing, respectively. The Q Exactive 2.8 SP 1 software (Thermo Fisher Scientific) was used to control the mass spectrometer.

For TMN, the Agilent GC/MS system (Agilent, Santa Clara, CA, United States) was used, because it is an apolar aromatic compound that could not be ionized for the detection by the UPLC-Q Exactive Orbitrap-HRMS system. The Agilent GC/MS system consisted of Agilent 3800 GC coupled to an Agilent Ion Trap 2200 MS/MS operated in electron impact ionization mode. The GC system was equipped with an electronic pressure control and an isothermal injector. One μL of cleaned extract was injected on a DB-5 column (30 m × 0.25 mm × 0.25 μm) using splitless injection mode. The injection temperature was set at 250°C. The GC temperature program was 60°C, hold 2 min, ramp 30°C/min to 120°C, ramp 10°C/min to 240°C, ramp 30°C/min to 300°C, and hold 5 min. Helium was used as carrier gas with a flow rate of 1.2 ml/min. The MS was employed in Multiple Reaction Monitoring mode. The ion source, ion trap, and interface temperatures were set at 200, 200, and 280°C. The results were quantified using Agilent MS Workstation software (v6.9.3). Recoveries of compounds were within 80–120%.

### In Silico Blood–Brain Barrier Permeability Prediction

The Ligand Express (Cyclica; Toronto, Ontario, Canada), admetSAR, and Biovia ADMET Blood Brain Barrier Model (Dassault 545 Systémes, Vélizy-Villacoublay, France) were used to predict the BBB permeability of the compounds. Ligand Express® is a cloud-based platform that screens small-molecule drugs against repositories of structurally characterized proteins or “proteomes” to determine polypharmacological profiles. In terms of BBB permeability prediction, the system implements a classification model based on machine learning methods using a compiled BBB dataset of 1335 BBB-permeable and 360 BBB-impermeable compounds. The Anatomical Therapeutic Chemical Classification was used to filter out compounds that have an ambiguous status regarding their passage through the BBB or were not strictly CNS-active. In addition, 45 molecules that are known to cross the BBB and 91 P-glycoprotein substrates on the BBB impermeable set were included (Seelig, 1998; Adenot and Lahana, 2004; Shen et al., 2010).

The admetSAR (v2.0) server was developed as a comprehensive source and free tool for *in silico* prediction of chemical absorption, distribution, metabolism, excretion, and toxicity (ADMET) properties on the basis of structure–activity relationships (Cheng et al., 2012; Yang et al., 2019). More than 40 predictive models are implemented in admetSAR for *in silico* filtering of new chemical ADMET properties. These models are trained by state-of-the-art machine learning methods. The BBB model was developed by using a similar dataset as that used by Ligand Express®, derived mainly from the work of Shen et al. which included 1839 compounds (1438 BBB-permeable and 401 BBB-impermeable compounds) (Shen et al., 2010). Because both of these platforms gave almost identical BBB penetration probability values for all compounds, only the results from Ligand Express® are shown in Table 1. Values equal to or close to 1 indicate compounds with a high probability of BBB penetration.

**TABLE 1:** Summary of MAO activities and BBB permeability prediction.

Compounds	MW (g/mol)	MAO a IC_50_ (μM)^a^	MAO B IC_50_ (μM)^a^	BBB Ligand Express^®^	Log (BB) Biovia ADMET^b^
Harmane	182	0.05 ± 0.03	>100	0.955	High
Norharmane	168	1.05 ± 0.36	9.27 ± 5.8	0.990	High
TIQ	133	33.04 ± 7.56	50.25 ± 9.99	0.996	High
TMN	200	1.14 ± 0.74	7.14 ± 2.29	0.912	High
Clorgyline	272	0.01 ± 0.01	3.93 ± 1.00	1.000	Very high
Pargyline	159	1.84 ± 0.79	1.07 ± 0.48	1.000	Very high

a Data are presented in mean ± SEM.

b Low < -0.52; medium = -0.52 to 0; high = 0 to 0.7; very high = > 0.7.

Abbreviations: ADMET = absorption, distribution, metabolism, excretion, and toxicity; BB = blood brain; BBB = blood-brain barrier; MAO = monoamine oxidase; MW = molecular weight; TIQ = 1.2,3,4-tetrahydroisoquinoline; TMN = 2,3,6-trimethyl-1,4-naphthoquinone.

Lastly, the Biovia ADMET Blood Brain Barrier Model was used to predict the BBB penetration of a molecule, defined as the ratio of concentrations (brain concentration/blood concentration) after oral administration, and to report the predicted penetration as well as a classification of penetration level. The model combines a confidence ellipse in the Polar surface area and LogP descriptor space, derived from over 800 orally administered compounds classified as CNS therapeutics with a robust regression model based on 120 compounds with measured penetration to predict Log (Brain Blood (BB)) penetration values for those molecules falling within the confidence ellipse (Egan et al., 2000). The model predicts the BB permeation level based on the categories “very high” (BB ratio >5:1), “high” (between 1:1 and 5:1), “medium” (between 0.3:1 and 1:1), “low” (<0.3:1), and “undefined” (outside the 99% confidence range ellipse). This translates into the regression model prediction values of Log (BB) > 0.7 for “very high”, 0 < Log (BB) < 0.7 for “high”, −0.52 < Log (BB) < 0 for “medium”, and Log (BB) < −0.52 for “low”. No prediction was made for compounds outside the 95% confidence ellipsoids.

### Statistics

The behavioral findings were analyzed in 1 min bins using two-way repeated measures ANOVA with Dunnett *post hoc* analysis. The analyses were conducted by using GraphPad Prism v8.2.1 (GraphPad Software, San Diego, CA, United States).

## Results

### Effects of the Compounds on Zebrafish NTT Response

**FIGURE 2:**
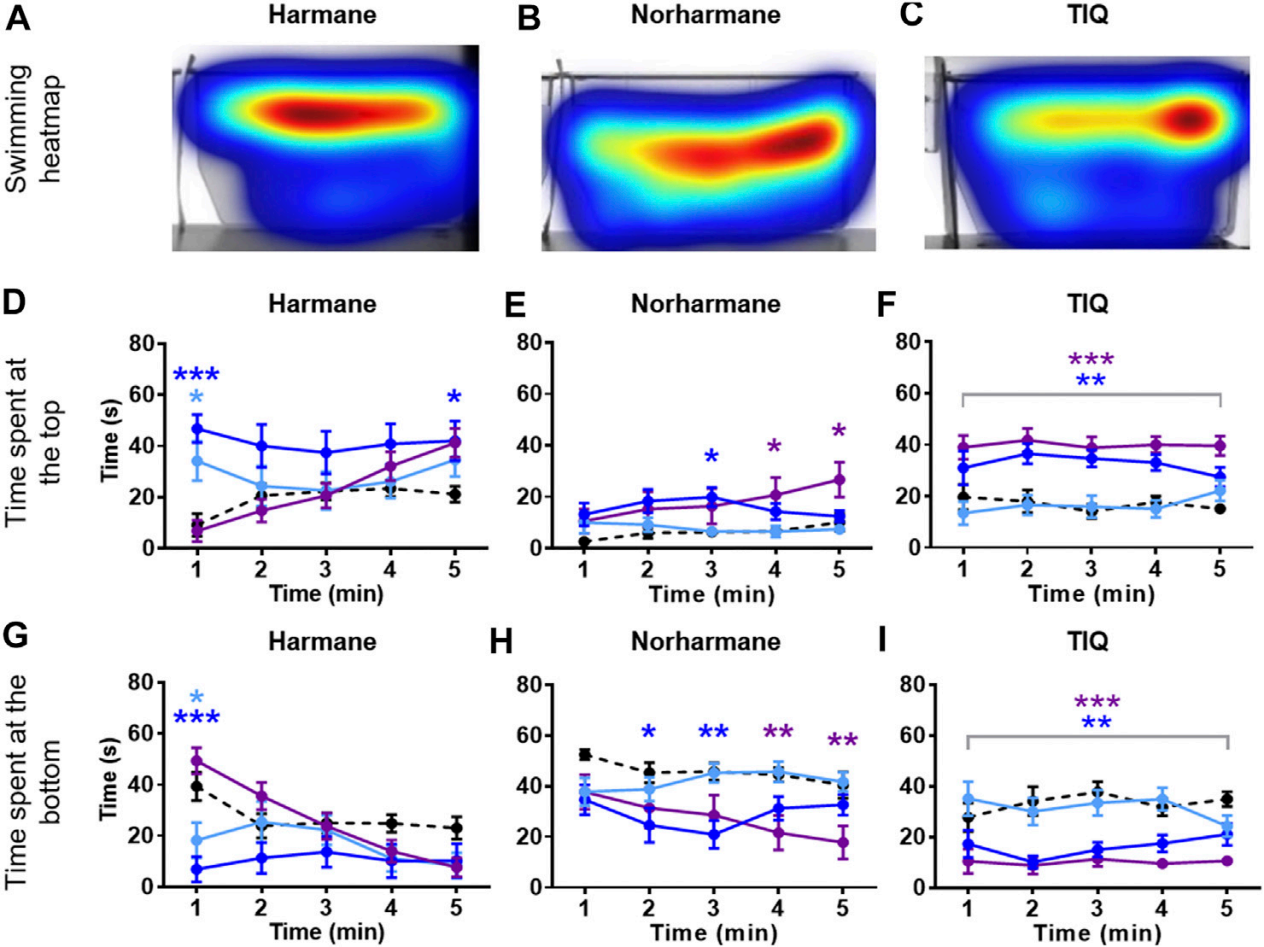
NTT results for harmane, norharmane, and TIQ. Representative heatmaps for **(A)** harmane (3 mg/L) **(B)** norharmane (3 mg/L), and **(C)** TIQ (100 mg/L) during the NTT for one zebrafish per compound are shown. The heatmaps show the minimum (dark blue) to maximum (dark red) amount of time a fish spent in each pixel. Time spent at the top and the bottom of the tank are quantified for **(D and G)** harmane (1, 3, and 10 mg/L), **(E and H)** norharmane (0.3, 1, and 3 mg/L), and **(F and I)** TIQ (10, 30, and 100 mg/L). All compounds show anxiolytic-like effects at at least one of the concentrations and time point. Black dashed line = vehicle control; light blue = lowest concentration; blue = middle concentration; purple = highest concentration. The sample size was 12 except for 10 mg/L harmane, control group for norharmane, 3 mg/L norharmane, and 100 mg/L TIQ, which were *n* = 11. Data are expressed as mean ± SEM. *p < 0.05; **p < 0.01; ***p < 0.001; Abbreviations: NTT = novel tank test; TIQ = 1.2,3,4-tetrahydroisoquinoline.

**FIGURE 3:**
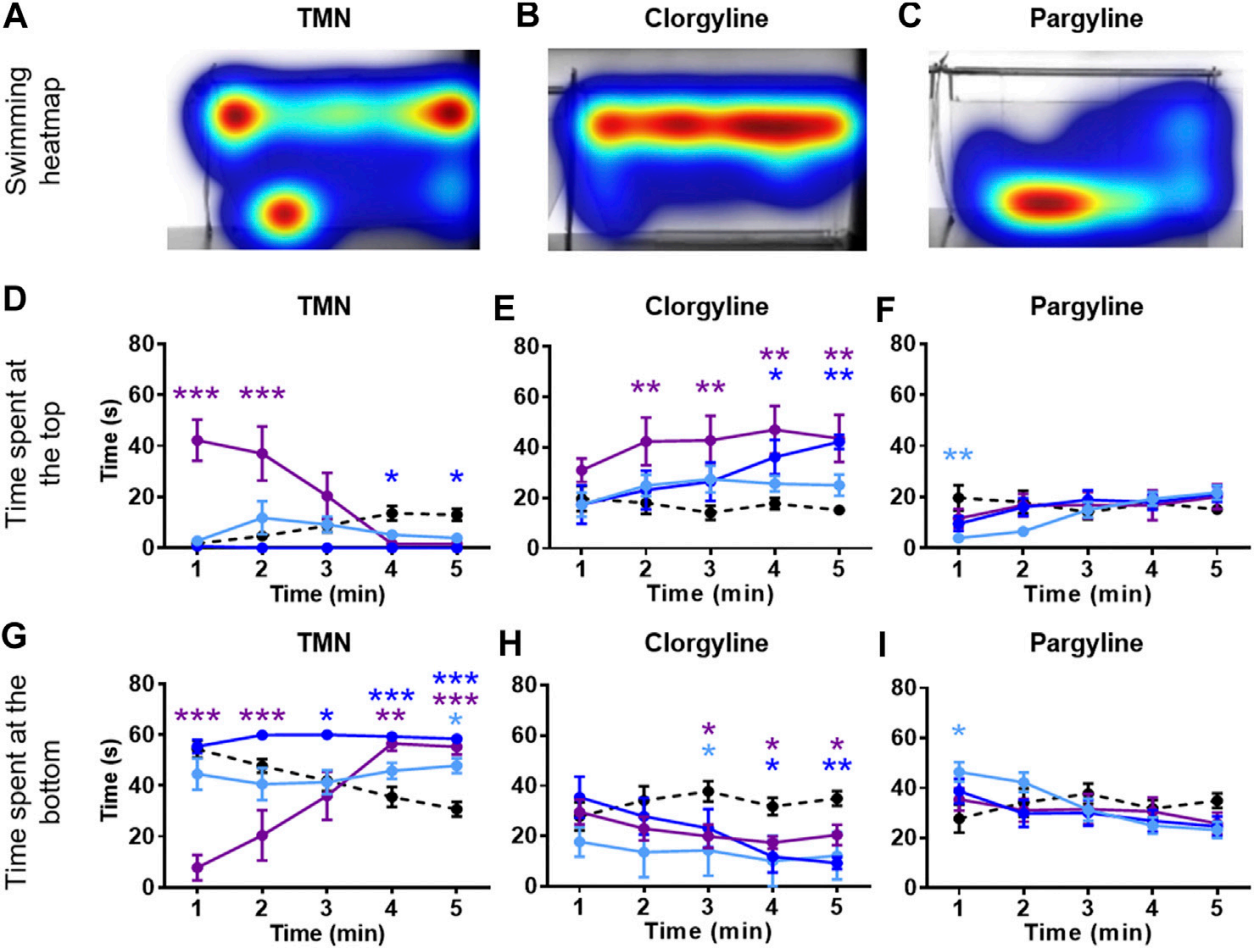
NTT results for TMN, clorgyline, and pargyline. Representative heatmaps for **(A)** TMN (30 mg/L) **(B)** clorgyline (100 mg/L), and **(C)** pargyline (10 mg/L) during the NTT for one zebrafish per compound are shown. The heatmaps show the minimum (dark blue) to maximum (dark red) amount of time a fish spent in each pixel. Time spent at the top and the bottom of the tank are quantified for **(D and G)** TMN (3, 10, and 30 mg/L), **(E and H)** clorgyline (10, 30, and 100 mg/L), and **(F and I)** pargyline (10, 30, and 100 mg/L). All compounds show anxiolytic-like effects at at least one of the concentrations and time point. Black dashed line = vehicle control; light blue = lowest concentration; blue = middle concentration; purple = highest concentration. The sample size was 12 except for control group for TMN (*n* = 11), 3 mg/L TMN (*n* = 9), 10 mg/L and 30 mg/L TMN (*n* = 7), 30 mg/L Clorgyline (*n* = 8), and 100 mg/L Clorgyline (n = 6). Data are expressed as mean ± SEM. *p < 0.05; **p < 0.01; ***p < 0.001; Abbreviations: NTT = novel tank test; TMN = 2,3,6-trimethyl-1,4-naphthoquinone.

Zebrafish were placed in an NTT tank immediately after being exposed to the system water containing one of the four natural MAO inhibitors (harmane, norharmane, TIQ, or TMN) or reference compounds (clorgyline or pargyline) for 20 min (Figure 1B). Interestingly, all four compounds induced a distinct behavioral profile in the zebrafish (Figures 2, 3; **Supplementary Figure S1** in Supplementary Material S1). There was a significant treatment effect by harmane (F (3, 43) = 5.311, *p* = 0.020 (top); F (3,43) = 4.047, *p* = 0.013 (bottom)). The lowest two concentrations of harmane (1 and 3 mg/L) increased the time spent at the top and decreased the time spent at the bottom during the first minute of the 5 min test (*p* < 0.05 for 1 mg/L; *p* < 0.001 for 3 mg/L), while the highest concentration (10 mg/L) had no effect (Figures 2A,D,G; treatment x time effect: F (12, 172) = 3.534, *p* = 0.0001 (top), F (12, 172) = 3.822, *p* < 0.0001 (bottom)). For norharmane, the highest concentration (3 mg/L) increased the time spent at the top and decreased the time spent at the bottom during the last 2 min of the test (*p* < 0.05 for top; *p* < 0.01 for bottom), while the middle concentration (1 mg/L) had a significant effect between 2 and 3 min of the test, particularly for the time spent at the bottom (*p* < 0.05 at 2 min; *p* < 0.01 at 3 min) (Figures 2B,E,H; treatment x time effect: F (12, 168) = 2.612, *p* = 0.0032 (top), F (12, 168) = 2.357, *p* = 0.0080 (bottom)). The lowest concentration (0.3 mg/L) had no effect. TIQ had a significant treatment effect also, where the highest two concentrations of TIQ (30 and 100 mg/L) generally increased and decreased the time spent at the top and bottom, respectively (*p* < 0.01 for 30 mg/L; *p* < 0.001 for 100 mg/L), while the lowest concentration had no effect (Figures 2C,F,I; F (3, 43) = 14.89, *p* < 0.0001 (top), F (3, 43) = 11.73, *p* < 0.0001). There was no time-dependent treatment effect. TMN induced a mix of both anxiolytic- and anxiogenic-like behaviors at the highest concentration tested (30 mg/L) (Figures 3A,D,G; treatment effect: F (3,30) = 8.746, *p* = 0.0003 (top); F (3, 30) = 7.128, *p* = 0.0009; treatment x time effect: F (12, 120) = 11.99, *p* < 0.0001 (top), F (12, 120) = 15.44, *p* < 0.0001 (bottom)). It increased the time spent at the top and decreased the time spent at the bottom during the first 2 min of the test (*p* < 0.001). In contrast, the fish spent more time at the bottom (*p* < 0.001) and had the tendency to spend less time at the top during the last 2 min of the test. The middle concentration (10 mg/L) of TMN induced only an anxiogenic-like effect during the last couple of minutes of the test (*p* < 0.05 for top; *p* < 0.001 for bottom).

Zebrafish treated with the two reference compounds, clorgyline and pargyline, also behaved quite differently from each other and from those treated with the natural MAO inhibitors. Clorgyline, in general, showed a concentration-dependent anxiolytic-like effect, with the highest concentration (100 mg/L) increasing the time spent at the top for almost the entire test period (*p* < 0.01), the middle concentration (30 mg/L) significantly increasing it only during the last 2 min (*p* < 0.05), and the lowest concentration (10 mg/L) having no effect (Figures 3B,E; treatment effect: F (3,34) = 4.864, *p* = 0.0064; treatment x time effect: F (12, 135) = 2.269, *p* = 0.0118). A corresponding reduction in the time spent at the bottom was evident, but the concentration-dependency was less prominent (Figure 3H; treatment effect: F (3, 34) = 3.225, *p* = 0.0345; treatment x time effect: F (12, 135) = 2.230, *p* = 0.0135). Pargyline was the only compound to show only an anxiogenic-like effect, but only for the first 1 min of the test at the lowest concentration tested (10 mg/L; *p* < 0.01 (top); *p* < 0.05 (bottom)) (Figures 3C,F,I; treatment x time effect: F (12, 176) = 3.520, *p* = 0.0001 (top); F (12, 176) = 3.400, *p* = 0.0002 (bottom)). It is worthy to note that buspirone, a clinical anxiolytic drug for general anxiety, induced a significant treatment effect in this paradigm (**Supplementary Figure S2** in Supplementary Material S1; treatment effect: F (3, 70) = 27.25, *p* < 0.0001 (top); F (3, 70) = 16.34, *p* < 0.0001 (bottom); time x treatment effect: F (12, 280) = 3.838, *p* < 0.0001 (top); F (12, 280) = 4.030, *p* < 0.0001 (bottom)). This finding supports the validity of the zebrafish NTT to detect an anxiolytic-like drugs.

**FIGURE 4:**
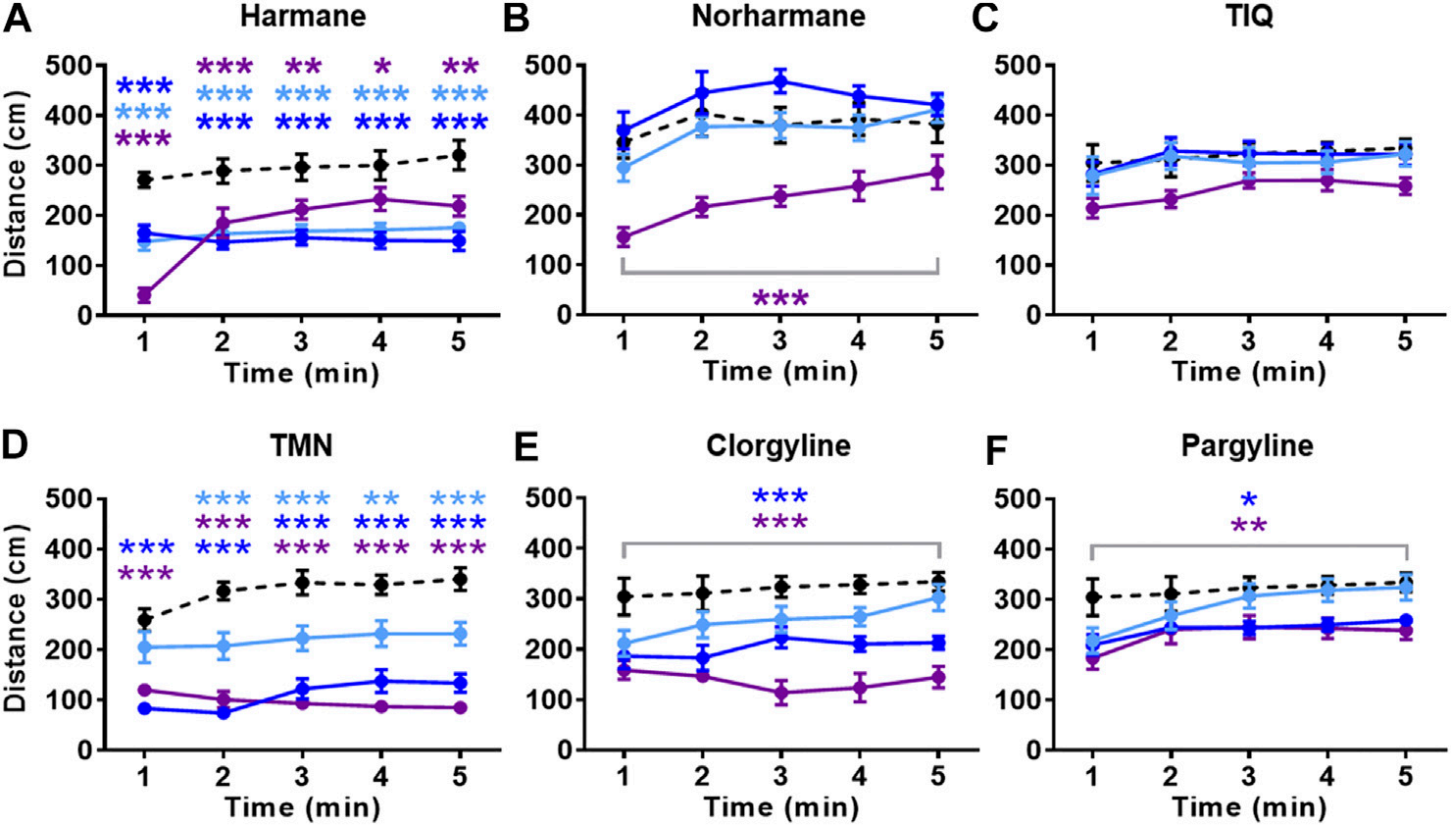
Effects of MAO inhibitors on total distance traveled. Total distance traveled during the NTT is presented for **(A)** harmane (1, 3, and 10 mg/L) **(B)** norharmane (0.3, 1, and 3 mg/L) **(C)** TIQ (10, 30, and 100 mg/L) **(D)** TMN (3, 10, and 30 mg/L) **(E)** clorgyline (10, 30, and 100 mg/L), and **(F)** pargyline (10, 30, and 100 mg/L). Black dashed line = vehicle control; light blue = lowest concentration; blue = middle concentration; purple = highest concentration. The sample size is detailed in the legends for Figures 3, 4. Data are expressed as mean ± SEM. *p < 0.05; **p < 0.01; ***p < 0.001. Abbreviations: NTT = novel tank test; TIQ = 1.2,3,4-tetrahydroisoquinoline; TMN = 2,3,6-trimethyl-1,4-naphthoquinone.

**FIGURE 5:**
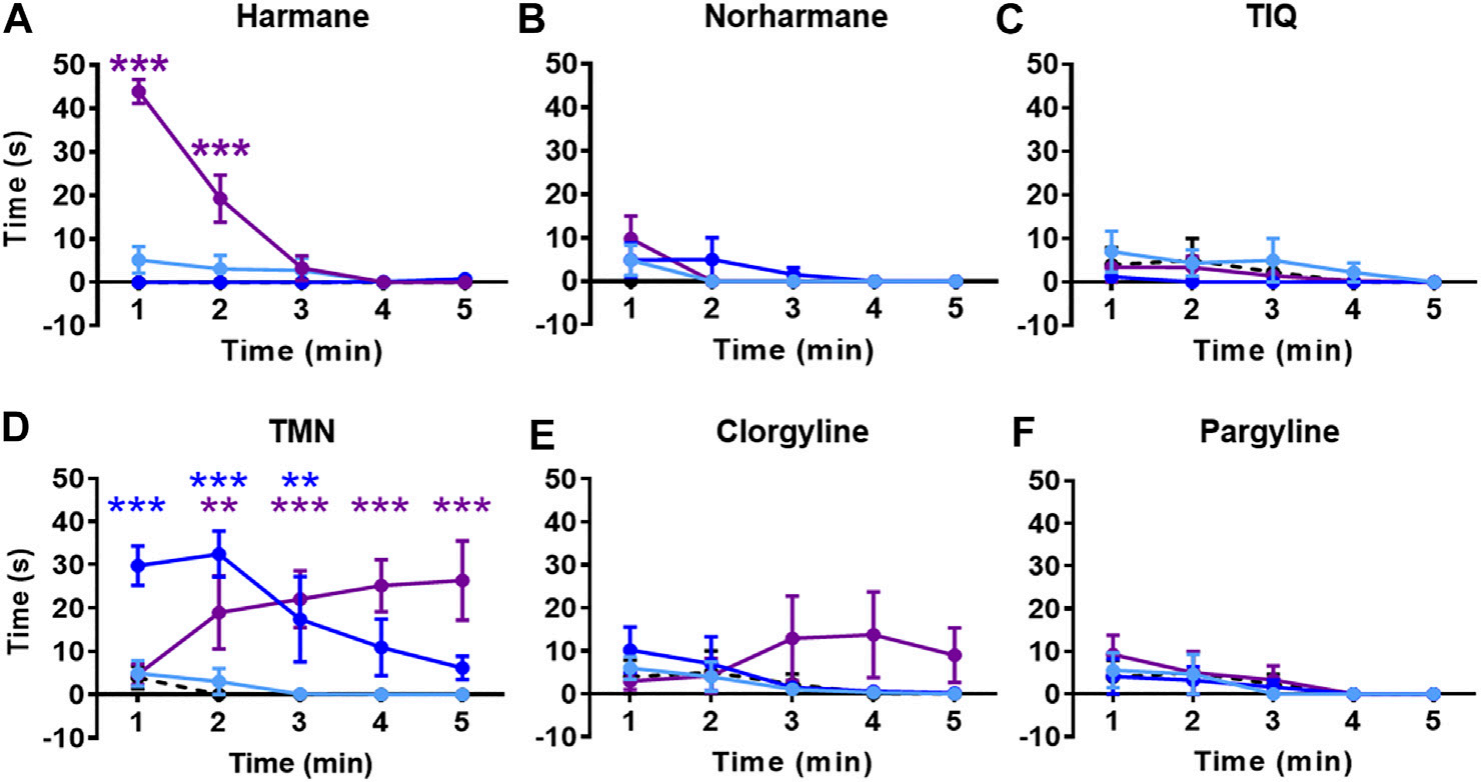
Effects of MAO inhibitors on freezing time. Total freezing time during the NTT is presented for **(A)** harmane (1, 3, and 10 mg/L) **(B)** norharmane (0.3, 1, and 3 mg/L) **(C)** TIQ (10, 30, and 100 mg/L) **(D)** TMN (3, 10, and 30 mg/L) **(E)** clorgyline (10, 30, and 100 mg/L), and **(F)** pargyline (10, 30, and 100 mg/L). Black dashed line = vehicle control; light blue = lowest concentration; blue = middle concentration; purple = highest concentration. The sample size is detailed in the legends for Figures 3, 4. Data are expressed as mean ± SEM. **p < 0.01; ***p < 0.001. Abbreviations: NTT = novel tank test; TIQ = 1.2,3,4-tetrahydroisoquinoline; TMN = 2,3,6-trimethyl-1,4-naphthoquinone.

All compounds except TIQ significantly affected the general activity, measured by total distance traveled (Figure 4). The total distance traveled was significantly reduced by all concentrations of harmane and TMN, the highest concentration of norharmane, and the two highest concentrations of clorgyline and pargyline throughout the test period (treatment effect for harmane: F (3, 43) = 16.81, *p* < 0.0001; for norharmane: F (3, 42) = 11.56, *p* < 0.0001; for TMN: F (3, 30) = 36.21, *p* < 0.0001; for clorgyline: F (3, 34) = 12.64, *p* < 0.0001; for pargyline: F (3, 44) = 4.656, *p* = 0.0065; treatment x time effect for harmane: F (12, 172) = 8.170, *p* < 0.0001; for TMN: F (12, 120) = 2.501, *p* = 0.0058). In addition, the highest concentration of harmane increased the freezing time during the first 2 min of the test (*p* < 0.0001) (Figure 5A; treatment effect: F (3, 43) = 23.94, *p* < 0.0001; treatment x time effect: F (12, 172) = 35.38, *p* < 0.0001). TMN increased the freezing time for the two highest concentrations (F (3, 30) = 15.50, *p* < 0.0001). The effect of the middle concentration was significant during the first 3 min (*p* < 0.01), while the effect of the highest concentration was significant from 1 min onward (*p* < 0.001) (Figure 5D; treatment x time effect: F (12, 120) = 4.920, *p* < 0.0001). Other compounds had no effect on the freezing response. The effects of the compounds on the total distance traveled and freezing time did not seem to parallel the anxiolytic- or anxiogenic-like effects of the respective compounds. Thus, it is not likely that any changes in the observed general movement had a direct association with the anxiolytic- or anxiogenic-like effects of the compounds.

### Monoamine Oxidase Inhibition

All compounds were tested for their efficacy to inhibit MAO-A and MAO-B by MAO assays *in vitro*. Of the four alkaloids, harmane was the most potent and selective MAO-A inhibitor in our assay (IC_50_ = 0.05 ± 0.03 and >100 µM for MAO-A and MAO-B, respectively) (Table 1). In fact, harmane was as potent as clorgyline in inhibiting MAO-A and showed greater MAO-A selectivity than clorgyline with >2,000-fold greater selectivity over MAO-B in contrast to the approximately 400-fold greater selectivity of clorgyline. Norharmane and TMN were approximately 100-fold less potent, and TIQ was 400-fold less potent than harmane (MAO-A IC_50_ = 1.05 ± 0.36 for norharmane, 1.14 ± 0.74 for TMN, and 20.88 ± 2.97 for TIQ). These three compounds did not show selectivity between MAO-A and MAO-B. The potency and lack of selectivity of norharmane and TMN most closely resembled those of pargyline.

### Blood–Brain Barrier Penetration

To understand whether the MAO inhibitors in the present study have good BBB penetrability, two qualitative classification models for predicting the probability of BBB penetration (Ligand Express® and admetSAR) and one quantitative regression model for predicting the Log (BB) compound penetration values (Biovia ADMET) were used. Both the Ligand Express® and admetSAR models predicted all compounds to have good BBB penetrability, as indicated by a probability close to 1 (Table 1). The predicted Log (BB) values for all compounds were in the high (0–0.7) or very high (>0.7) range. To confirm the predicted BBB penetration values, the concentrations of the compounds in brain were tested in bioavailability assay. All compounds were detected at high concentrations in the zebrafish brain (Table 2). Harmane and TIQ levels, in particular were approximately 10-fold higher than the rest of compounds tested. The observed concentrations for other compounds were within the expected level (1 ng/mg brain tissue), assuming that the brain density is approximately the same as that of water (Weisenburger and Vaziri, 2018).

**TABLE 2:** Summary of compound bioavailability in fish brain.

Compounds	Brain Analytes	Relative brain level per 1 mg/L compound^a^ ^,^ ^b^ (ng/mg tissue)	Log (BB) Biovia ADMET
Harmane	Harmane	10.64 ± 2.36	0.188
Norharmane	Norharmane	2.81 ± 1.52	0.101
TIQ	TIQ	11.47 ± 1.07	0.089
TMN	TMN	1.08 ± 0.33	0.268
Clorgyline	Clorgyline	3.92 ± 1.85	1.053
Pargyline	Pargyline	0.92 ± 0.37	0.750

a Data are presented in mean ± SEM.

b Relative compound level in the brain per 1 mg/L compound was calculated by assuming a linear relationship between compound concentration and BBB penetration.

Abbreviations: ADMET = absorption, distribution, metabolism, excretion, and toxicity; BB = blood brain; TIQ = 1,2,3,4-tetrahydroisoquinoline; TMN = 2,3,6-trimethyl-1,4-naphthoquinone.

## Discussion

MAO inhibitors have been previously reported to ameliorate depression and specific types of anxiety (Sabri and Saber-Ayad, 2020). In this study, we detected anxiolytic-like effects of four natural MAO inhibitors – harmane, norharmane, TMN, and TIQ – in novel-environment-induced anxiety by using the zebrafish NTT. The observed effects were most likely due to a direct regulation of brain function as all tested compounds showed a high level of brain bioavailability and predicted blood-brain-barrier permeability. The anxiolytic-like effect of harmane is in agreement with previous reports indicating reduction in anxiety-related behaviors in, for example, elevated plus maze in rodents (Aricioglu and Altunbas, 2003; Khan et al., 2017a). Such effect of norharmane has been speculated (Pepplinkhuizen et al., 1996), but it has not been reported. There have been no previous reports regarding the anxiolytic-like effect of TIQ or TMN. We believe that this is the first time that the anxiolytic-like effect of these compounds for this particular type of anxiety, as predicted in this zebrafish model, is reported. There are several different types of anxiety recognized in *Diagnostic and Statistical Manual of Mental Disorders* by American Psychiatric Association, including, for example generalized anxiety disorder, panic disorder, social anxiety disorder, and agoraphobia (Leahy et al., 2012; Salum et al., 2013; Park and Kim, 2020). The zebrafish NTT may most closely mimic agoraphobia, a type of panic disorder characterized by symptoms of anxiety in situations where the person perceives their environment to be unsafe with no easy way to escape. In this paradigm, a zebrafish fears the potential threat of predator in a new environment, and dives to the bottom of the tank, avoiding the potential danger. This behavior is interpreted as an anxiety-like response.

Classically, MAO inhibitors have been considered as a treatment of panic disorders with agoraphobia, but their use was rather restricted due to the risk of, for example, hypertension (Tyrer and Shawcross, 1988; Vina et al., 2012; Sabri and Saber-Ayad, 2020). The alkaloids in this study, however, belong to the reversible type of MAO inhibitors, which have been favored due to reduced side effects (Buller, 1995; Herraiz and Chaparro, 2005; Wasik et al., 2014). It is worthy to note that three out of four test compounds also reduced the total distance travelled at the concentrations that showed anxiolytic-like effect in zebrafish. TIQ was the only compound that showed a steady anxiolytic-like effect throughout the test period without affecting the general movement of the fish. The reason for the observed difference may be difficult to interpret purely on the basis of MAO-A or -B activities. For example, TIQ was the weakest MAO-A/B inhibitor *in vitro*, yet it induced the most steady anxiolytic-like effect during the behavioral test. Its brain bioavailability and blood-brain barrier permeability probability were similar to harmane. One alternative explanation is that these naturally occurring compounds have other molecular targets in addition to MAO. For example, previous reports suggest that harmane and norharmane can also bind benzodiazepine receptor binding albeit at high concentrations (Muller et al., 1981). We have also observed serotonin receptor and norepinephrine transporter binding activities for harmane and norharmane in the 10 µM range in a pilot study *in vitro* (data not shown). Thus, it is possible that the anxiolytic-like effect of TIQ may be mediated by other molecules in addition to MAO.

It is worth noting that in the present study, clorgyline induced anxiolytic-like effect while pargyline did not. This finding supports and confirms previous reports indicating that the MAO A inhibitor clorgyline more effectively regulates mood and anxiety than the MAO B inhibitor pargyline (Tyrer and Shawcross, 1988). In support of this behavioral finding, we found that, in fact, clorgyline inhibits MAO A approximately 400-fold stronger than MAO B, which is similar to previous reports by others suggesting approximately 1000-fold selectivity (Ramsay et al., 2020). The *in vitro* inhibitory activity of pargyline was less straight forward to interpret, where we detected similar IC_50_ values – thus, similar Ki values (0.85 μM for MAO A and 0.50 μM) – for MAO A and B. This is in line with the previous observation by Fisar et al. (2010), showing 1.4-fold difference between the Ki values of MAO-A and MAO-B by using MAO from crude pig brain mitochondrial fractions (Fisar et al., 2010). Interestingly, it has been shown that assay conditions (e.g., preincubation time) can affect the apparent selectivity of pargyline (Fowler et al., 1982; Ramsay et al., 2020). For example, with the direct assay without substrate preincubation with pargyline, the Ki values were 15 μM for MAO A and 1.8 μM for MAO B (Fowler et al., 1982; Ramsay et al., 2020), yielding a mere 8-fold difference which cannot be considered as a selectivity. In contrast, recent studies performed by Oh et al. (2020) and Takao et al. (2019) observed >100-fold and 21-fold differences in Ki values of MAO-A and MAO-B, respectively, by using recombinant human MAO-A and MAO-B enzymes (Oh et al., 2020) (Takao et al., 2019). One could speculate that additional differences in, for example, pargyline batch or salt form, substrate type, MAO enzyme type or source, and enzymatic reaction condition may have contributed to the slightly different MAO selectivity observed for pargyline in the present study and by others. This new pharmacological insight suggests that perhaps, previous conclusion that the lack of anxiolytic-like effect by pargyline is due solely to the lack of MAO A inhibitory activity may be over simplistic and should be interpreted with caution. The MAO inhibitory potency and selectivity of the other four compounds tested in this study – harmane, norharmane, TIQ, and TMN – were within the range of previous reports substrate (Thull et al., 1995; Herraiz and Chaparro, 2005; Dos Santos Passos et al., 2014; Wiart, 2014). Of these compounds, harmane was the only MAO A selective inhibitor, while norharmane, TIQ, and TMN showed no selectivity between MAO A and B in the present study and in previous reports (Thull et al., 1995; Herraiz and Chaparro, 2005; Coelho Cerqueira et al., 2011; Dos Santos Passos et al., 2014). Because TIQ, TMN, and norharmane were non-selective MAO inhibitor similar to pargyline, it is possible that the anxiolytic-like effect observed for TIQ, TMN, and norharmane may be partially induced by other molecular targets in addition to MAO.

In conclusion, anxiety disorders are among the most common psychiatric disorders that affect all groups of the general population. The current available treatments have unwanted side effects, such as excessive sedation, cognitive impairment, ataxia, aggression, sexual dysfunction, tolerance and dependence (Vina et al., 2012). In this study, we have shown that MAO-inhibiting compounds that are naturally present in plants can induce anxiolytic-like effects in zebrafish. TIQ, in particular, showed a promising neurobehavioral profile, inducing steady anxiolytic-like effect without affecting the general movement. The current findings highlight the importance of investigating natural compounds as alternative herbal remedies for anxiety and support the usefulness of zebrafish as an experimental tool to screen for anxiolytic-like compounds.

## Data Availability

The raw data supporting the conclusion of this article will be made available by the authors, without undue reservation.
