# Supplementary material for: Effects of Natural Monoamine Oxidase Inhibitors on Anxiety-Like Behavior in Zebrafish
Source: Front Pharmacol. 2021 May 13;12:669370. doi: 10.3389/fphar.2021.669370 (PMC8165606; doi:10.3389/fphar.2021.669370)
Supplement: Supplementary file 1 [file datasheet1.pdf]

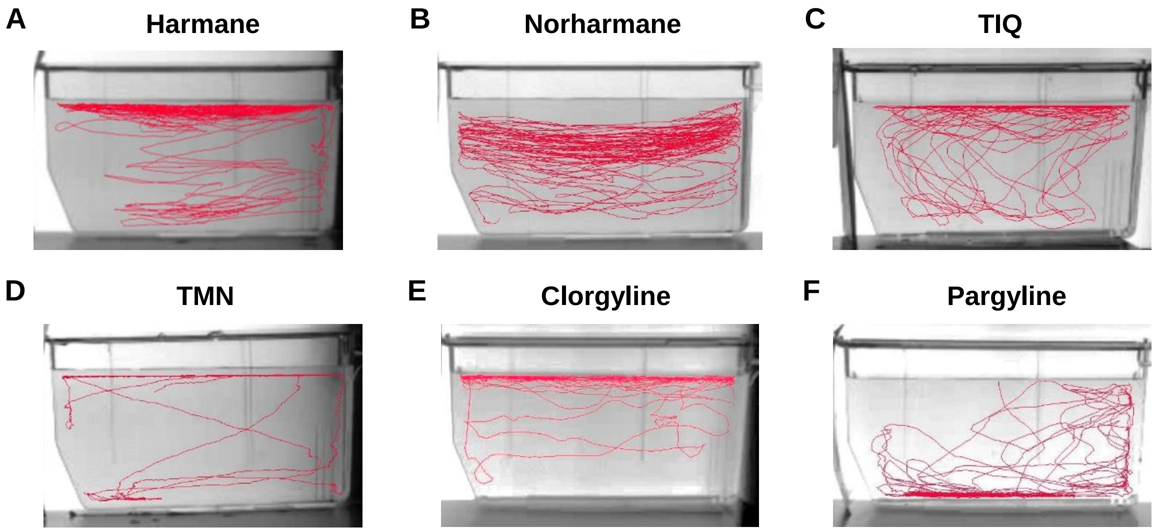

Supplementary Figure 1. Representative swimming traces
Corresponding swimming traces for heat maps in **Figs. 2A-C** and **3A-C** are presented for (A) harmane (3 mg/L), (B) norharmane (3 mg/L), (C) TIQ (100 mg/L), (D) TMN (30 mg/L), (E) clorgyline (100 mg/L), and (F) pargyline (10 mg/L), representing swimming behavior for one zebrafish per compound. Data are expressed as mean ± SEM. * $p < 0.05$; ** $p < 0.01$; *** $p < 0.001$.

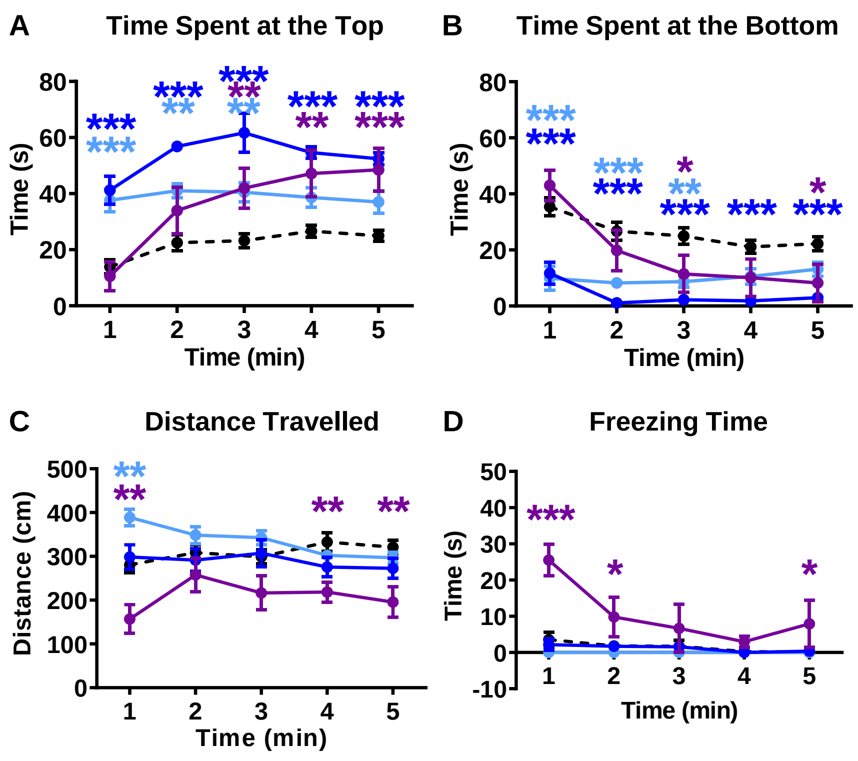

Supplementary Figure 2. Zebrafish NTT results for buspirone
(A) Time spent at the top, (B) time spent at the bottom, (C) total distance travelled, and (D) freezing time are presented for buspirone. Black dashed line = control (n = 35); light blue = 10 mg/L buspirone (n = 12); blue = 30 mg/L buspirone (n = 18); purple = 100 mg/L buspirone (n = 9). Data are expressed as mean ± SEM. * $p < 0.05$; ** $p < 0.01$; *** $p < 0.001$.
